# Early Immune Responses in Rainbow Trout Liver upon Viral Hemorrhagic Septicemia Virus (VHSV) Infection

**DOI:** 10.1371/journal.pone.0111084

**Published:** 2014-10-22

**Authors:** Rosario Castro, Beatriz Abós, Jaime Pignatelli, Louise von Gersdorff Jørgensen, Aitor González Granja, Kurt Buchmann, Carolina Tafalla

**Affiliations:** 1 Centro de Investigación en Sanidad Animal (CISA-INIA), Valdeolmos (Madrid), Spain; 2 Department of Veterinary Disease Biology, Faculty of Health and Medical Sciences, University of Copenhagen, Frederiksberg, Denmark; Universitat de Barcelona, Spain

## Abstract

Among the essential metabolic functions of the liver, in mammals, a role as mediator of systemic and local innate immunity has also been reported. Although the presence of an important leukocyte population in mammalian liver is well documented, the characterization of leukocyte populations in the teleost liver has been only scarcely addressed. In the current work, we have confirmed the presence of IgM^+^, IgD^+^, IgT^+^, CD8α^+^, CD3^+^ cells, and cells expressing major histocompatibility complex (MHC-II) in rainbow trout (*Oncorhynchus mykiss*) liver by flow cytometry and/or immunohistochemistry analysis. Additionally, the effect of viral hemorrhagic septicemia virus (VHSV) on the liver immune response was assessed. First, we studied the effect of viral intraperitoneal injection on the transcription of a wide selection of immune genes at days 1, 2 and 5 post-infection. These included a group of leukocyte markers genes, pattern recognition receptors (PRRs), chemokines, chemokine receptor genes, and other genes involved in the early immune response and in acute phase reaction. Our results indicate that T lymphocytes play a key role in the initial response to VHSV in the liver, since CD3, CD8, CD4, perforin, Mx and interferon (IFN) transcription levels were up-regulated in response to VHSV. Consequently, flow cytometry analysis of CD8α^+^ cells in liver and spleen at day 5 post-infection revealed a decrease in the number of CD8α^+^ cells in the spleen and an increased population in the liver. No differences were found however in the percentages of B lymphocyte (IgM^+^ or IgD^+^) populations. In addition, a strong up-regulation in the transcription levels of several PRRs and chemokines was observed from the second day of infection, indicating an important role of these factors in the response of the liver to viral infections.

## Introduction

Among the essential functions of the liver, including carbohydrate, protein and lipid metabolism, bile secretion and detoxification, its role as a mediator of systemic and local immunity points to this organ as an important site for immune regulation. In fact, the liver is a primary hematopoietic organ in mammals during the fetal stage. Before the fetal liver develops, definitive hematopoietic progenitors committed to B, T or myeloid lineages, emerge in the aorta-gonad-mesonephros region early in gestation (day 10) [1], [2]. Immediately after, the liver is colonized by these committed progenitors and this tissue becomes a primary hematopoietic organ during prenatal stages [3]. Then, the bone marrow gradually replaces the liver as a primary hematopoietic organ until the time of birth when it takes over completely. However, the postnatal liver retains a substantial population of immunologically active cells with specific functional and phenotypic characteristics. Hepatic cells of myeloid lineage, including Kupffer cells and dendritic cells, and intrahepatic lymphocytes including B and T cells, natural killer (NK) cells and NK cells expressing T cell receptor (NKT cells) have been described in mammalian liver [4], [5]. In addition, its anatomical structure and extensive vasculature imply the exposition to a continuous traffic of immune information. The liver receives a dual blood supply, on one hand, a large volume of blood enters from the gastrointestinal tract through the portal vein, carrying a wide number of orally ingested antigens; while on the other hand, oxygenated blood coming from the systemic circulation enters via the hepatic artery, providing information on the systemic immune responses [6]. Thus, the liver has to be at the same time an immunocompetent organ while controlling inappropriate inflammatory reactions provoked by harmless dietary antigens [7]. And therefore, the induction of tolerance to ingested and self-antigens must be precisely regulated [8]. Among its immune functions the liver has a key role in non-specific phagocytosis and cell killing, production of acute phase proteins and complement, removal of pathogens, antigens and molecules systemically produced during inflammation. Functions related with specific immunity are also part of the hepatic system, including extrathymic proliferation of T cells, deletion of activated T cells and specific immune-regulation [8].

In teleost fish, very little attention has been paid to the immunological role of the adult liver. For decades, evidences revealed the presence of an important population of intraparenchymal macrophages in the liver of different fish species [9]–[12], but the existence of additional resident leukocyte subtypes in teleost liver has only recently been described for rainbow trout (*Oncorhynchus mykiss*) [13]. This study reported the presence of a resident leukocyte population accounting for 15–29% of the non-hepatocytes in the liver. Using several pan-specific antibodies the authors suggested that these cells included T and B lymphocytes and myeloid cells, with predominance of B cells (38%) over T cells (13%) [13]. In mammals however, the hepatic lymphocyte population is dominated by T cells, NK cells and NKT cells, whereas B cells represent a small subpopulation within the liver [14].

In the current work, we have undertaken a close examination of the trout hepatic lymphocyte subpopulation of cells by both flow cytometry and immunohistochemistry using specific monoclonal antibodies against rainbow trout IgM, IgT, IgD, CD8α and MHC-II. Additionally, we have studied the immune response of the liver to a systemic viral infection with viral hemorrhagic septicemia virus (VHSV), a novirhabdovirus responsible for a lethal disease in many cultivated fish species worldwide, including rainbow trout [15]–[17]. We have analyzed the transcriptional responses of the liver to the viral infection, including a group of leukocyte markers genes, genes belonging to the pattern recognition receptor system (PRR), chemokines and chemokine receptor genes, and other genes involved in the early immune response and acute phase reactions. Furthermore, changes in leukocyte populations have also been studied in response to the infection. Our results point to the liver as an important immune site involved in the response to VHSV infection, which seems mainly orchestrated at the initial phase of the response by T cells.

## Materials and Methods

### Ethics statement

The experiments described comply with the Guidelines of the European Union Council (2010/63/EU) for the use of laboratory animals and were previously approved by the Ethics committee from the Instituto Nacional de Investigación y Tecnología Agraria y Alimentaria (INIA; Code CEEA 2011/044). Anesthesia was applied prior to sacrifice following the recommendations from Zhal *et al*. for general anesthesia (narcosis) [18]. All efforts were focused to minimize suffering.

### Fish

Female rainbow trout (*Oncorhynchus mykiss*) of approximately 30–50 g obtained from Centro de Acuicultura El Molino (Madrid, Spain) were maintained at the animal facilities of the Centro de Investigación en Sanidad Animal (CISA-INIA, Spain) in a re-circulating water system at 16°C, with 12:12 hours Light:Dark photoperiod. Fish were fed twice a day with a commercial diet (Skretting, Spain). Prior to any experimental procedure, fish were acclimatized to laboratory conditions for at least 2 weeks.

### Flow cytometry analysis of resident leukocyte populations in unstimulated liver

The distribution of leukocyte populations in the liver of naïve fish was analyzed in blood depleted (buffer perfused) animals. For this, fish were anesthetized with 30 mg/L benzocaine in water [18]. Subsequently, a transcardial perfusion was conducted to remove the circulating blood from the tissues. The heart was cannulated through the ventricle into the bulbus arteriosus for perfusion with 30 ml of teleost Ringer solution pH 7.4, with 0.1% procaine, using a peristaltic pump at a constant flow rate of ∼5 ml/min, whereas the atrium was cut to drain the blood out of the circulatory system. After perfusion, the liver was sampled and placed in L-15 with 100 I.U./ml penicillin, 100 µg/ml streptomycin (P/S), 10 units/ml heparin and 2% FCS, pushed through a 100 µm nylon mesh and the resulting cell suspensions were placed onto a 30/51% discontinuous Percoll gradient, and centrifuged at 500×g for 30 min at 4°C. The interface cells were collected, washed at 500×g for 5 min in L-15 containing 0.1% FCS and resuspended in L-15 with P/S, and 2% FCS. For comparative purposes, spleen and whole kidney were sampled in parallel.

The leukocyte suspensions obtained were adjusted to a final concentration of 1×10^6^ cells/ml and incubated with the monoclonal antibodies (mAbs) against trout immune cell markers in staining buffer (PBS with 1% FBS and 0.1% sodium azide) for 20 min at 4°C. The mAbs used were anti-trout IgM (mAb 1.14 mouse IgG, coupled to phycoerythrin, 0.1 µg/ml) [19], anti-trout IgD (mAb mouse IgG, 5 µg/ml) [20], anti-trout MHC-II β-chain (mAb mouse IgG, coupled to biotin 2 µg/ml), anti-trout CD8α (mAb rat IgG, 7 µg/ml) [21] and anti-trout thrombocytes (mAb mouse IgG, coupled to phycoerythrin, 0.1 µg/ml) [13]. After the incubation, cells were washed twice with staining buffer and secondary Abs/conjugates were added when applied as follows: the secondary Ab for anti-IgD mAb was an allophycocyanin croslinked F(ab')_2_ fragment goat anti-mouse (H+L) (Invitrogen), the secondary Ab for anti-CD8α detection was a R-phycoerythrin F(ab')_2_ fragment of goat anti-rat IgG (H+L) (Invitrogen) and the conjugate for the anti-MHC-II mAb was FITC-streptavidin (BD Biosciences). After 20 min of incubation and three washes, cells were analyzed on a FACSCalibur flow cytometer (BD Biosciences).

### RNA extraction and cDNA preparation

Total RNA was extracted from liver samples using a combination of Trizol (Invitrogen) and RNAeasy Mini kit (Qiagen) previously described [22]. In summary, samples were mechanically disrupted in 1 ml of Trizol using a disruption pestle. Then, 200 µl of chloroform were added and the suspension centrifuged at 12000×*g* for 15 min. The clear upper phase was recovered, mixed with an equal volume of 100% ethanol and immediately transferred to RNAeasy Mini kit columns. The procedure was then continued following manufacturer's instructions, performing on-column DNase treatment. Finally, RNA pellets were eluted from the columns in RNase-free water, quantified in a Nanodrop 1000 spectrophotometer (Thermo Scientific) and stored at −80°C until used. Two μg of RNA were used to obtain cDNA in each sample using the Bioscript reverse transcriptase (Bioline Reagents Ltd) and oligo (dT)_12-18_ (0.5 µg/ml) following manufacturer's instructions. The resulting cDNA was diluted in a 1∶5 proportion with water and stored at −20°C.

### Evaluation of immune gene expression by real time PCR

To evaluate the levels of transcription of the different genes, real-time PCR was performed in a LightCycler 480 System instrument (Roche) using SYBR Green PCR core Reagents (Applied Biosystems) and specific primers (shown in Fig. S1). The efficiency of the amplification was determined for each primer pair using serial 10 fold dilutions of pooled cDNA, and only primer pairs with efficiencies between 1.95 and 2 were used. Each sample was measured in duplicate under the following conditions: 10 min at 95°C, followed by 40 amplification cycles (15 s at 95°C and 1 min at 60°C). The expression of individual genes was normalized to relative expression of trout EF-1α and the expression levels were calculated using the 2^−ΔCt^ method, where ΔCt is determined by subtracting the EF-1α value from the target Ct. Negative controls with no template were included in all the experiments. A melting curve for each PCR was determined by reading fluorescence every degree between 60°C and 95°C to ensure only a single product had been amplified.

### VHSV *in vivo* infection

Rainbow trout of approximately 50 g were divided into two groups of 22 fish and injected intraperitoneally (i.p.) with 100 µl of either 5×10^5^ TCID_50_/ml of the VHSV strain 0771, or the same volume of PBS. At days 1, 2 and 5 post-infection, six trout from each group were sacrificed by over-exposure to MS-222. The liver was extracted and placed in Trizol to be processed for RNA extraction and real time PCR as above. At day 5, four additional fish per group where sampled for FACS analysis as follows: the liver was placed in L-15 with P/S, 10 units/ml heparin and 2% FCS, pushed through a 100 µm nylon mesh and separated onto a Percoll gradient as above. The infection and sampling procedures were repeated in two independent experiments.

### Immunohistochemistry

Excised livers from control and infected fish were fixed in Bouin's solution for 24 h and processed to be embedded in paraffin (Paraplast Plus; Sherwood Medical) and sectioned at 5 µm. After dewaxing and rehydration, sections were subjected to an indirect immunocytochemical method to detect the different trout leukocyte markers. After a heat induced epitope retrieval in Tris-EDTA buffer pH 9.0 (800 w for 5 min and 450 w for 5 min in a microwave oven), the sections were pre-incubated in a blocking solution consisting of 2% BSA (bovine serum albumin; Sigma-Aldrich) in TBT (Tris buffer with 0.2% tween 20) at room temperature for 10 min, and 10% normal goat serum in TBT for 10 min. Then sections were incubated with primary antibody solution overnight at 4°C. Mouse anti-IgM and anti-IgT mAbs previously described [23], [24] (Fig. S2) were used at a concentration of 10 µg/ml. Anti-trout IgD and anti-trout MHC-II mAbs were used at concentrations of 10 µg/ml and 4 µg/ml respectively. Because the anti-trout CD8α mAb used in flow cytometry does not work in immunohistochemistry, in this case we used a mAb against trout CD3 that effectively works in immunohistochemistry, kindly provided by Dr. Erin Bromage from the University of Massachusetts Dartmouth (USA) [25], [26]. Following this incubation, unbound primary antibodies were washed off using TBT. The tissue was covered with Dako REAL detection System, alkaline phosphatase/RED, Rabbit/mouse (Dako) biotinilated secondary antibody and following manufacturer's instructions for staining. The specificity of the reactions was determined by omitting the primary antibodies. Mayer's haematoxylin (Dako) was used as nuclear counter stain, and mounting was conducted with Aquamount (Merck). Slides were examined with an Axiolab (Zeiss) light microscope.

### Statistics

Data handling, analyses and graphic representation was performed using Microsoft Office Excel 2010. Statistical analyses were performed using two tailed unpaired *t* Student's tests, after having confirmed that both populations (control and infected) did not display statistically different variances. Means ± standard deviation of the mean (SD) for each group were calculated.

## Results

### Characterization of resident leukocyte populations in unstimulated liver

**Figure 1:**
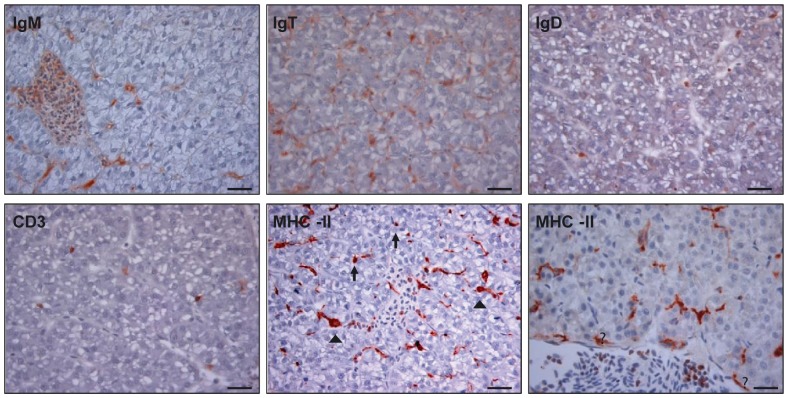
Imunohistochemical detection of different leukocyte populations in trout liver. Representative photomicrographs of anti-IgM, anti-IgD, anti-IgT, anti-CD3, and anti-MHC-II positive staining in liver sections obtained from unstimulated fish (N = 4). Different phenotypes were observed among MHC-II^+^ cells including small lymphocyte-like round cells (arrows) and macrophage/dendritic-like cells (arrow heads). Some of these MHC-II^+^ cells appear as part of the endothelial layer of the blood vessels (asterisks). Counterstained with Mayer's haematoxylin. Scale bar represents 20 µm.

Although the presence of IgM^+^ B cells in the liver was recently reported [13], [27], the presence of additional B and T cell populations has only been suggested. In the present work, we characterized the resident leukocyte population in unstimulated liver using mAb against specific leukocyte markers. The distribution of these leukocyte populations was studied by immunohistochemistry (IgM, IgD, IgT, MHC-II and CD3) and by flow cytometry (IgM, IgD, MHC-II and CD8α) using monoclonal antibodies previously characterized (Fig. S2) [21], [25], [28]. Through immunohistochemistry, as already reported [27], IgM^+^ cells were not only identified within the liver blood vessels but also homogeneously dispersed along the stroma of the liver, presenting a strong staining central core (Fig. 1). A similar distribution was observed for IgT^+^ cells. Some, less abundant, IgD^+^ and CD3^+^ cells were also observed dispersed along the parenchyma as small round cells with intense staining. MHC-II^+^ cells (that should mainly include B cells, dendritic cells and macrophages) are also homogeneously distributed along the liver, some of them with a lymphocyte-like morphology (small round cells with large nucleus), and other cells displaying a macrophage or dendritic-like morphology (big cells of irregular shape, with strong staining intensity in both the core of the cytoplasm and the prolongations of the cell). Interestingly, some of these MHC-II^+^ cells appear as part of the endothelial layer of the blood vessels (Fig. 1), in agreement with the antigen-presenting cell phenotype of mammalian hepatic endothelial cells, that includes the constitutive expression of MHC-II [29], [30].

**Figure 2:**
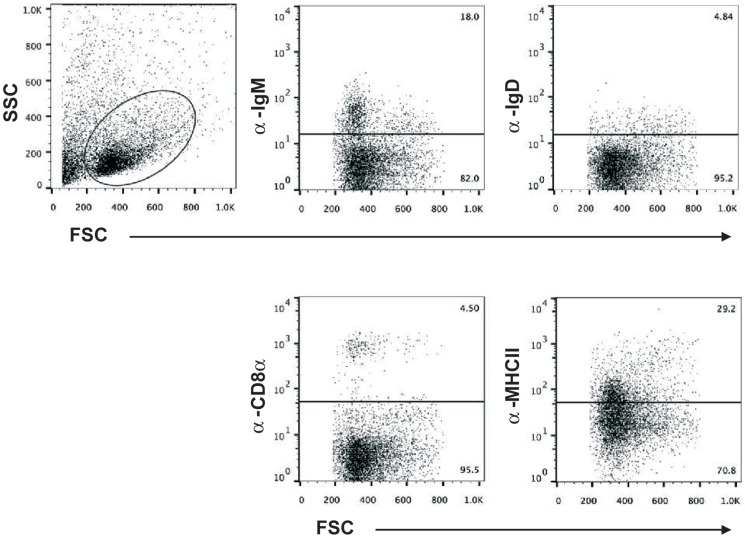
Flow cytometry analysis of leukocyte populations in trout liver. Distribution of leukocyte cells in rainbow trout liver tissue. Flow cytometry of rainbow trout liver leukocytes stained with anti-IgM, anti-IgD, anti-CD8α, and anti-MHC-II mAbs. First dot plot shows SSC/FSC profile of liver leukocytes. Subsequent plots show fluorescence intensity versus FSC of the gated cells.

Liver leukocyte populations from blood depleted perfused fish were also isolated using Percoll density gradient separation and characterized by flow cytometry. Discrete populations of B lymphocytes were observed among the leukocytes isolated from the liver, including cells bearing IgM (13.7±8.2% of the leukocyte gate) and IgD (2.8±1.6%) (Fig. 2, Table 1). About 25% of the total leukocyte population was positive for MHC-II, whereas CD8α^+^ cells represented approximately 4% of the resident leukocytes and a residual population of thrombocytes was observed (0.85%).

**Table 1:** Mean percentage of distinct leukocyte populations positive for different leukocyte markers in unstimulated perfused trout liver in comparison to spleen and kidney (N = 6).

Populations	Liver	Spleen	Kidney
IgM^+^	13.70±8.20	28.64±14.66	14.45±2.93
IgD^+^	2.83±1.60	3.86±1.95	2.67±1.96
MHC-II^+^	23.30±6.30	72.30±5.65	63.47±14.12
CD8α^+^	3.91±1.02	4.34±0.59	3.37±1.15
Thrombocytes^+^	0.85±0.40	5.84±4.54	0.80±0.29

The percentages were calculated over the leukocyte gate.

### Transcription of immune genes in blood-perfused liver

Prior to establishing the effects that VHSV had on the capacity of the liver to transcribe diverse immune genes, we studied the constitutive transcription levels of all of these immune related genes in liver samples obtained from perfused fish. The basal transcription levels were assessed by real time PCR and are compiled in Figure S3.

### Viral transcription in the liver of VHSV-infected trout

The effects of VHSV intraperitoneal injection on transcription of immune related genes in the liver was assessed at short time periods after the infection (days 1, 2 and 5). Before this study was conducted, we studied the levels of transcription of the G glycoprotein gene in the liver, as an indicator of viral replication levels in this tissue. At day 1 post-injection, G glycoprotein transcription levels remained undetected, however, they were already visualized at day 2 post-injection and they remained elevated at similar levels at day 5 post-injection (Figure S4).

### Effect of VHSV intraperitoneal infection on the transcription of leukocyte markers in the liver

**Figure 3:**
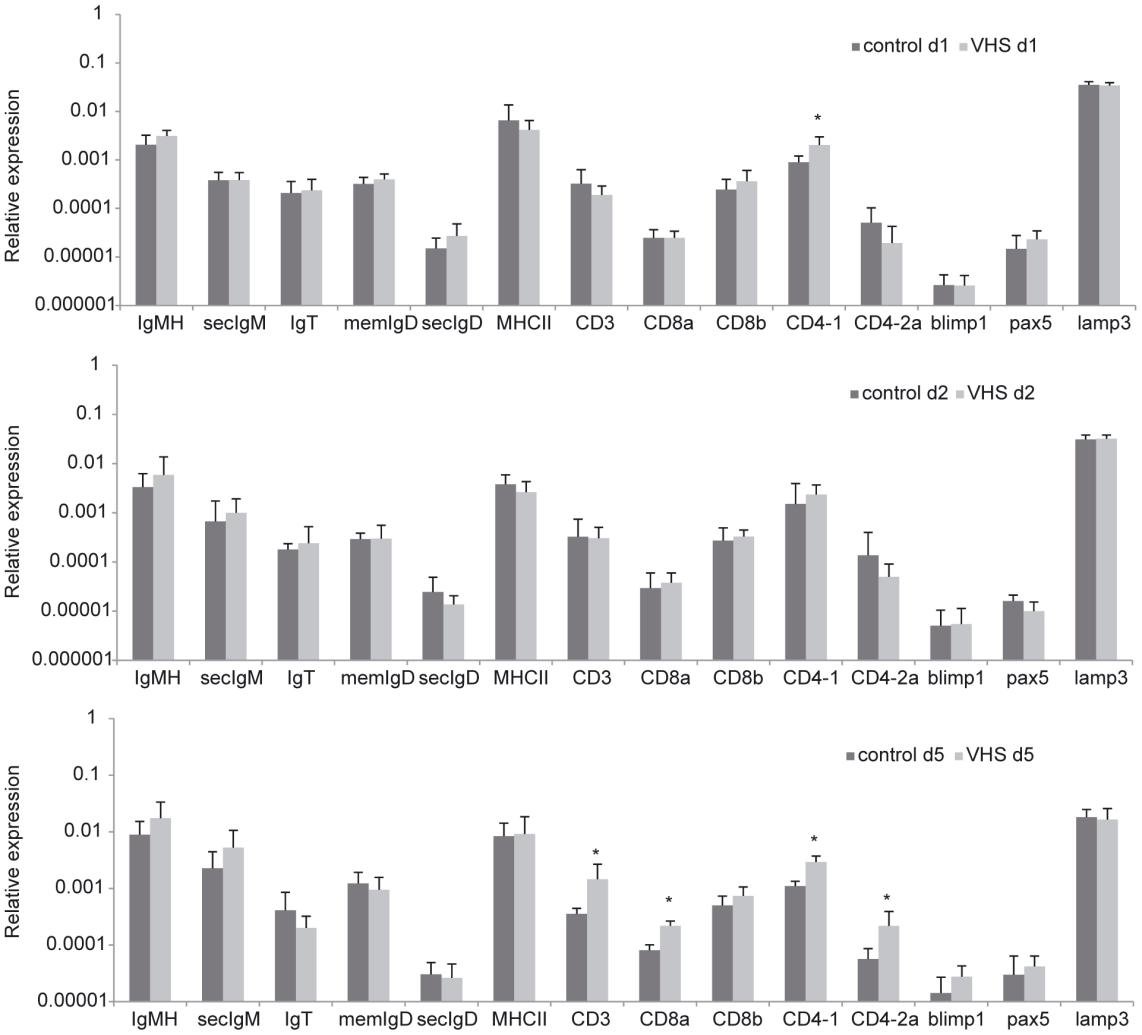
Transcription levels of immune genes characteristic of different leukocyte subpopulations in liver in response to VHSV. Rainbow trout were ip. infected with VHSV (5×10^5^ TCID_50_/ml) or mock-infected. At days 1, 3 and 5 post-infection six trout from each group were killed and the liver sampled to determine the levels of expression of a selection of immune genes by real-time PCR. Data are shown as the mean gene expression relative to the expression of endogenous control EF-1α ± SD. * Levels of expression significantly different to those observed in mock-infected fish (p<0.05).

Firstly, the transcription levels of B lymphocyte marker genes, including total membrane and secreted IgM heavy chain, secreted IgM, total IgT, membrane IgD and secreted IgD were analyzed by real time PCR. We could not detect an effect of viral challenge in the levels of mRNA transcription at neither of the time points studied. On the contrary, the anti-viral response in the liver seemed to be more directed to a T-dependent response, as we observed significant up-regulation of the transcription levels of several T lymphocyte markers including CD3, CD8α, CD4-1 and CD4-2 at day 5 post-challenge (Fig. 3). Other B lymphocyte markers such as Blimp1, Pax5, or markers related with antigen presentation cells like MHC-II or Lamp3 showed no significant regulation due to viral infection at the time periods studied.

### Effect of VHSV intraperitoneal infection on the transcription of pattern recognition receptors

**Figure 4:**
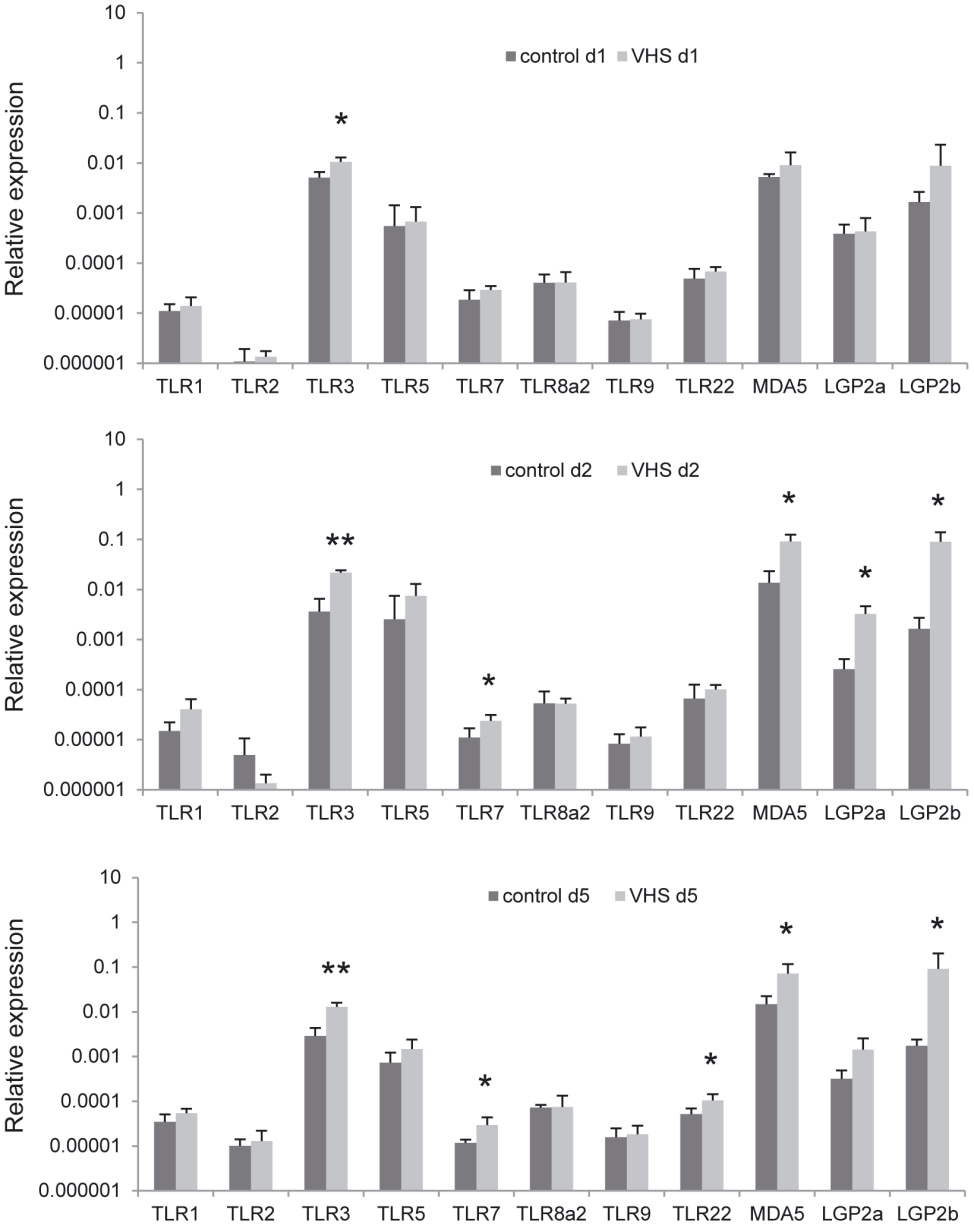
Transcription levels of PRRs genes in liver in response to VHSV. Rainbow trout were infected with VHSV as described in the legend of Figure 4 and the liver sampled to determine the levels of expression of PRR genes by real-time PCR. Data are shown as the mean gene expression relative to the expression of endogenous control EF-1α ± SD. Expression levels significantly different to those observed in mock-infected fish are noted as * (p<0.05), ** (p<0.001).

Initial virus recognition is mainly related with the PRR system, including TLRs and RIG I-like receptors [31], [32]. In our model, the TLR3 gene appears significantly induced from the first day post-viral injection (Fig. 4), and this induction is even more significant (p<0.0002) at days 2 and 5 post-infection. After 2 days, TLR7 is also significantly up-regulated, and this significance is maintained at 5 days. TLR22, a fish specific TLR that recognizes double stranded RNA on the cell surface [33], was also significantly induced in liver samples from infected fish after 5 days (Fig. 4). Transcription of genes related to the RIG I-like receptors system such as MDA5, LGP2a and LGP2b were also significantly triggered in samples from VHSV-challenged fish.

### Effect of VHSV intraperitoneal infection on the transcription of chemokines and chemokine receptors

**Figure 5:**
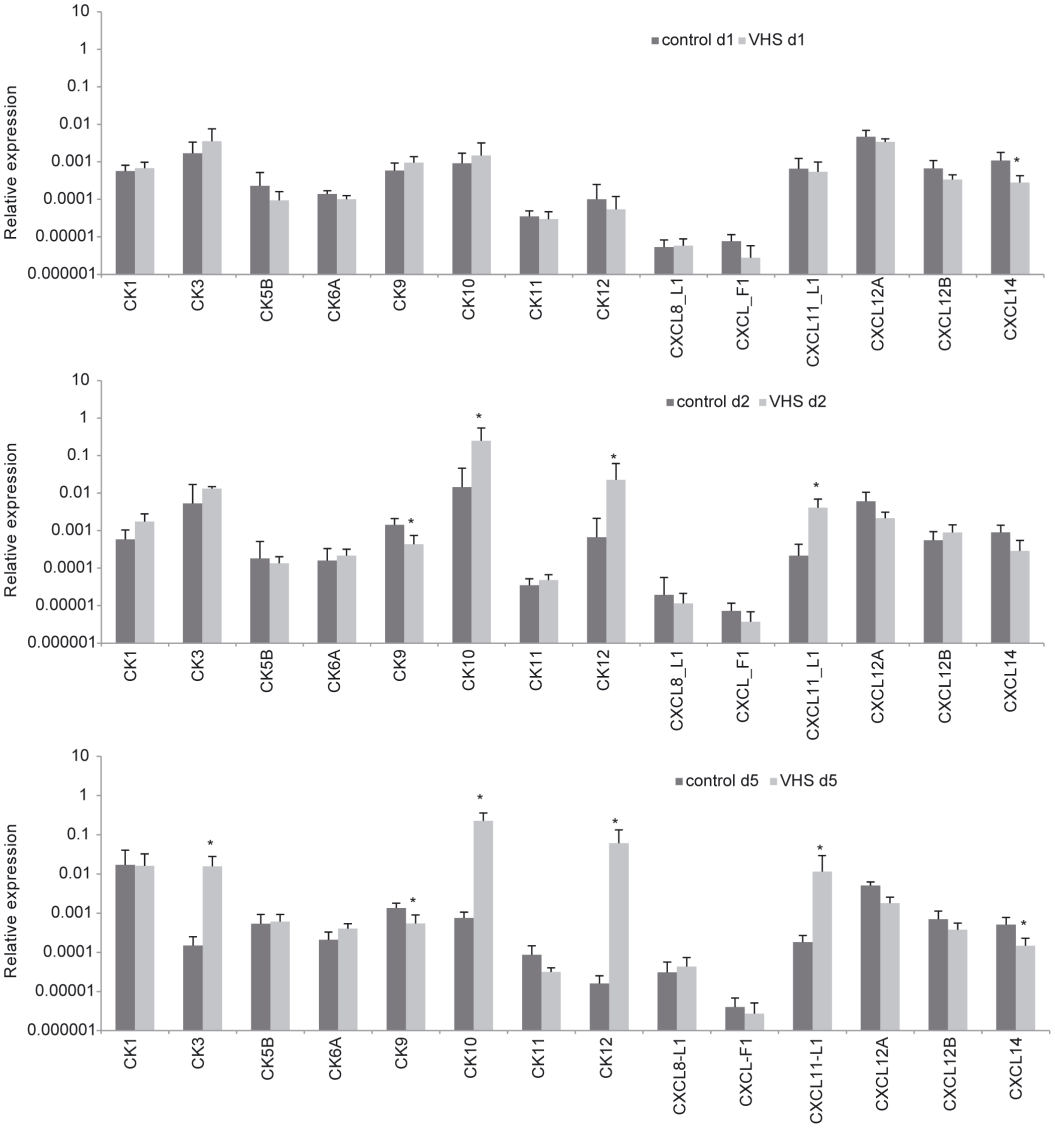
Chemokine transcription in liver after VHSV challenge. Rainbow trout were infected with VHSV as described in the legend of Fig. 4 and the liver sampled to determine the levels of expression of chemokines by real-time PCR. Data are shown as the mean gene expression relative to the expression of endogenous control EF-1α ± SD. * Expression levels significantly different to those observed in mock-infected fish (p<0.05).

Twenty four hours after VHSV challenge, the mRNA levels of the trout chemokines examined in liver were similar between infected and control samples, with the exception of a significant down-regulation of CXCL14 in response to the virus. After 2 days of infection, the transcription of CK10, CK12 and CXCL11_L1 was significantly up-regulated compared to controls, whereas CK9 mRNA levels were significantly down-regulated in infected fish (Fig. 5). At day 5 post-injection, CK10, CK12 and CXCL11_L1 transcription remained significantly increased in infected animals, in addition to CK3 which also appeared up-regulated at this time point. Again, the transcription of CK9 and in this case also CXCL14 was significantly down-regulated in livers from infected fish compared to controls.

**Figure 6:**
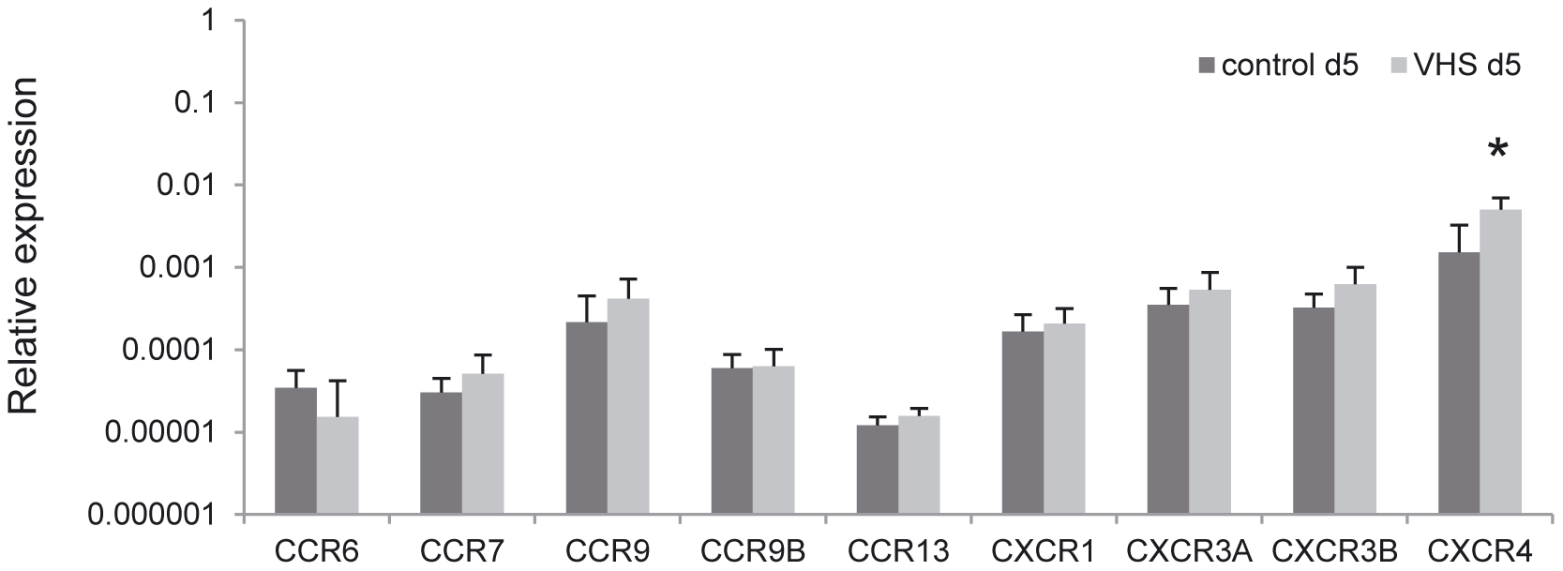
Chemokine receptor transcription in liver after VHSV challenge. Rainbow trout were infected with VHSV as described in the legend of Fig. 4 and the liver sampled to determine the levels of expression of chemokine receptor genes by real-time PCR. Data are shown as the mean gene expression relative to the expression of endogenous control EF-1α ± SD. * Expression levels significantly different to those observed in mock-infected fish (p<0.05).

In contrast with the strong regulation observed for some chemokines, only one chemokine receptor gene was differentially regulated in the liver in response to VHSV. CXCR4 transcription significantly increased in the liver of VHSV-infected fish compared to levels observed in liver obtained from control animals, but only after 5 days of infection (Fig. 6). These results might indicate that chemokine receptors are initially regulated at a protein level and not transcriptionally, or alternatively, that other non-described receptors are involved in the recognition of the up-regulated chemokines.

### Effect of VHSV intraperitoneal infection on the transcription of other genes related with early immune responses

**Figure 7:**
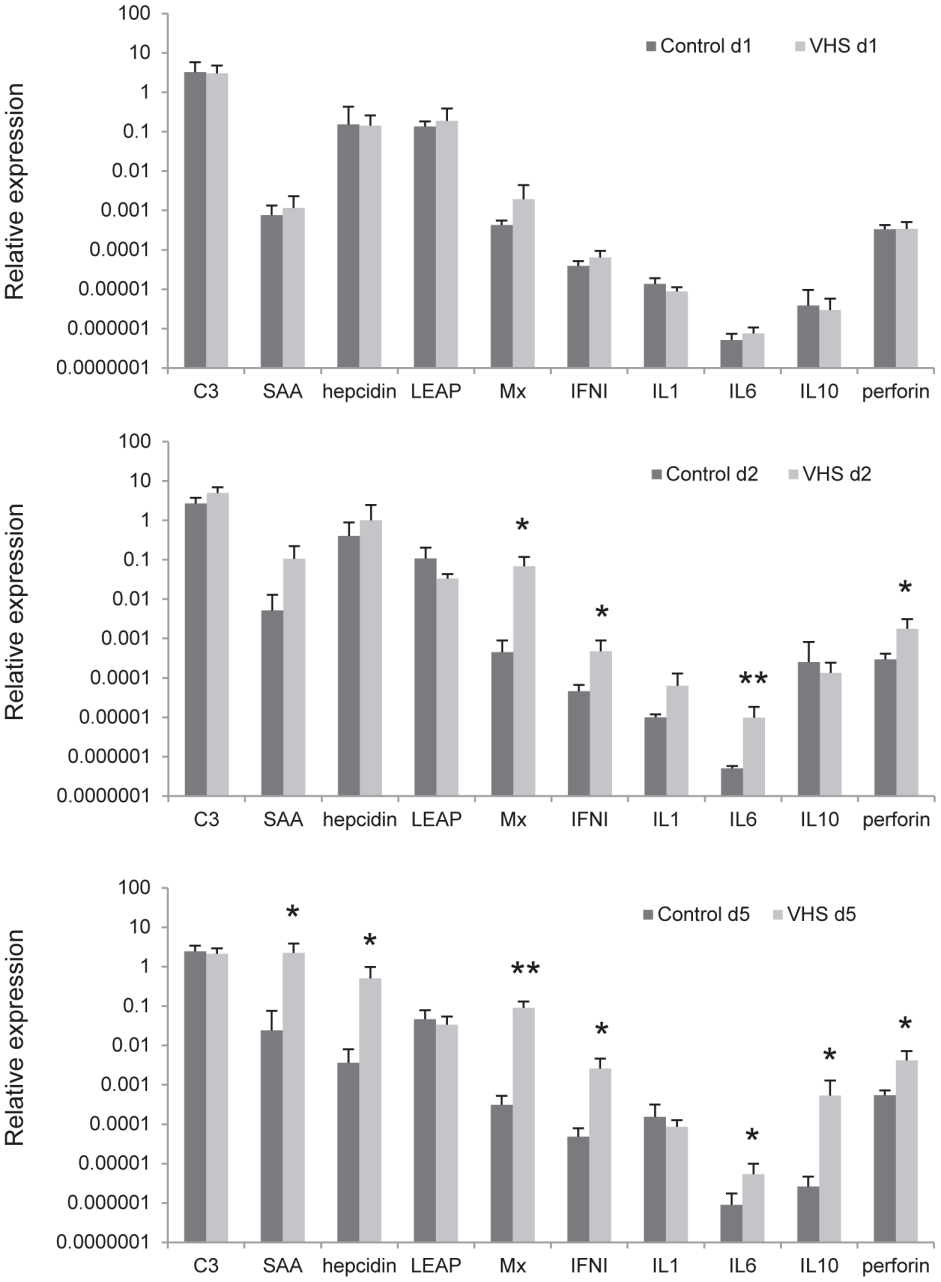
Transcription levels of other genes of the immune response/acute phase reaction in liver in response to VHSV. Rainbow trout were infected with VHSV as described in the legend of Fig. 4 and the liver sampled to determine the levels of expression of different genes related with the initial response of the immune system by real-time PCR. Data are shown as the mean gene expression relative to the expression of endogenous control EF-1α ± SD. Expression levels significantly different to those observed in mock-infected fish are noted as * (p<0.05), ** (p<0.001).

We have also studied the effect of VHSV on the transcription of other key genes involved in the early immune response and in the acute phase reactions. Complement factors and proteins of the acute phase response are mainly produced by the liver and can be transcriptionally regulated in fish in response to pathogens. SAA, for example, is a major acute phase protein showing up to 1000-fold increase in human plasma during inflammation and its hepatic synthesis is induced by inflammatory cytokines, mainly IL-1, IL-6 and TNF-α [34]. In our experiments, we observed an increased transcription of SAA at day 5 post-infection in response to VHSV that might be a consequence of the up-regulated IL-6 transcription detected from the second day of infection (Fig. 7). Hepcidin, another acute phase protein, was significantly up-regulated at day 5 post-infection, whereas no differences in the expression of LEAP-2 (liver-expressed antimicrobial peptide 2), the complement component C3 or IL-1β where found at any time point between control and infected fish. On the other hand, hallmark genes of the response against viral infection such as IFN1 or Mx where strongly up-regulated in the liver from day 2 (Fig. 7). An increase in IL-10 and perforin transcription was observed at day 5 and both day 2 and 5 respectively.

### Effect of VHSV intraperitoneal infection on lymphocyte percentages in the liver

**Figure 8:**
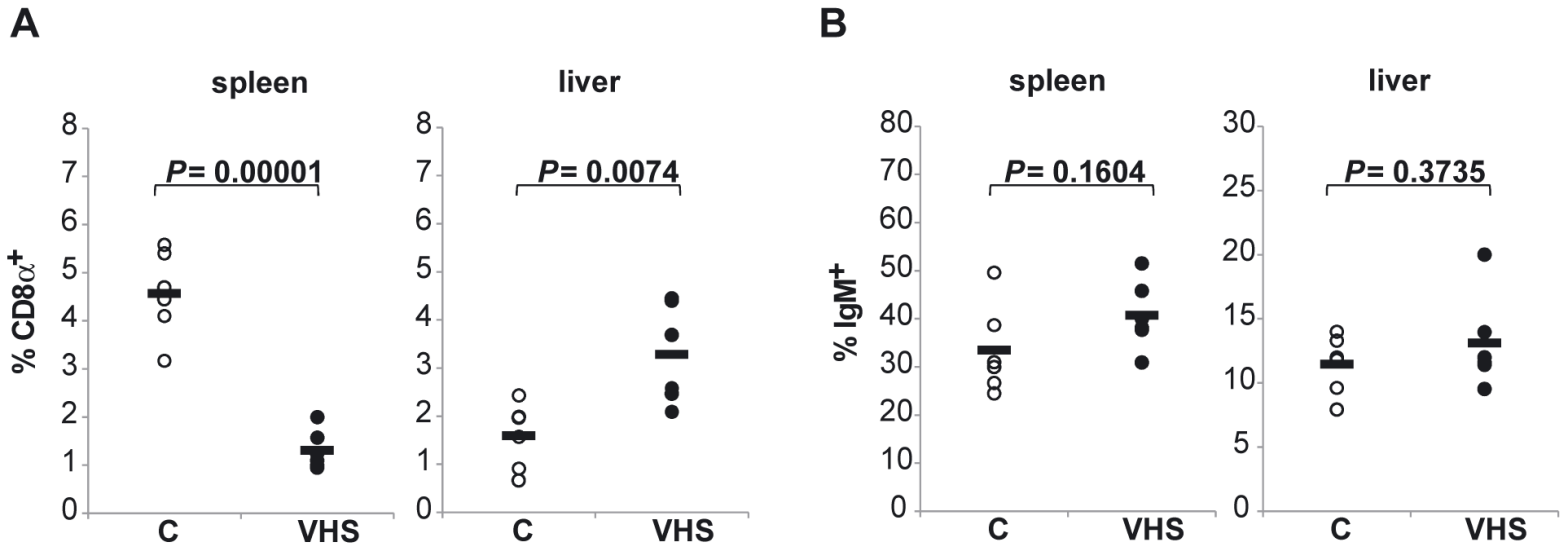
Distribution of CD8α^+^ and IgM^+^ cells in liver and spleen in response to a VHSV infection. The number of CD8α^+^ (A) and IgM^+^ (B) cells was determined through flow cytometry in spleen and liver of trout infected with VHSV at day 5 post-infection and compared to the number of cells obtained in mock-infected controls. Circles represent number of positive cells in individual fish, whereas black bars represent mean values in each experimental group.

We studied through immunohistochemistry whether VHSV provoked changes in the IgM, IgD, IgT, CD3 and MHC-II staining pattern in the liver, however through this methodology, no significant differences were observed (Figure S5). Thus, to further understand the immune response of the liver to viral infection, we analyzed the percentages of IgM^+^, IgD^+^, CD8α^+^ and MHC-II^+^ cells in the liver of control and infected animals through flow cytometry. We performed these studies at day 5 post-infection because this was the time point when most of the transcriptional changes occurred; furthermore, we compared the leukocyte numbers with those in the spleen. The analysis of leukocyte populations on liver leukocyte samples from control and infected fish showed a significant modification of CD8α^+^ ratios in both spleen and liver after 5 days of virus challenge (Fig. 8A). Interestingly, while CD8α^+^ cell ratios significantly diminished in spleen, they augmented in liver samples from infected fish compared to controls. On the other hand, no significant changes in the percentages on IgM^+^ cells were observed after 5 days post-injection on splenic or hepatic populations (Fig. 8B). No changes on IgD^+^ or MHC-II^+^ populations were detected either (data not shown).

## Discussion

The presence of lymphocytes in normal adult liver has been well documented in mammals. In humans, immunohistochemical staining of normal liver samples after extensive perfusion has demonstrated that these resident lymphocytes are predominantly located around the portal tracts and also distributed scattered throughout the parenchyma [4]. Similarly, in our studies we have detected the presence of different B cell subpopulations expressing IgM^+^, IgD^+^ or IgT^+^ on the cell surface, and CD3^+^ cells (T lymphocytes) dispersed within the parenchyma of healthy rainbow trout liver. Interestingly, the relative proportion of B/T cells into the hepatic leukocyte population described for rainbow trout is bend to a predominance of B cells over T cells [13] and these results seemed confirmed by our immunohistochemistry studies, since cell bearing CD3, an expected marker for all T cell populations [25], seemed less abundant than B cells. Furthermore our study demonstrates that this B cell population is heterogeneous, consisting in cells that bear different Ig isotypes in their membrane, namely IgM, IgT and IgD. This is the first description of IgT^+^ cells in the teleost liver, although unfortunately the mAb used does not work in flow cytometry. Teleost IgT^+^ B cells do not bear IgM on the cell surface and where first described as B cells with an important role in mucosal responses [35], even though posterior studies have seen their regulation outside mucosal tissues [22], [36]. In any case, it is worth noting that when a transcriptional characterization of IgM^+^ cells from different tissues was performed in rainbow trout, liver IgM^+^ cells had similar expression profiles to IgM^+^ cells from mucosal tissues such as gills or gut, different to the transcriptional profiles observed in IgM^+^ cells from immune organs such as the spleen or the head kidney [27]. This could be a consequence of liver B cells being exposed to a high antigenic load as B cells in mucosal tissues. In mammals, the majority of hepatic B cells are positive for CD5 cell surface glycoprotein, a pan T cell marker that also identifies a subtype of innate B cells known as B1 [5]. B1 cells use a restricted receptor gene segment, they can be activated in a T cell-independent way and produce low affinity autoreactive IgM antibodies referred to as natural antibodies [37], [38]. The polyreactivity of natural antibodies produced by CD5^+^ cells results in the recognition of autoreactive antigens and probably removal of apoptotic bodies. These innate properties of the B1 subset of B cells could be strongly related to the roles of the liver in immunological clearance and tolerance [5]. Notably, mammalian B1 cells are also characterized by very low levels of IgD in the cell surface [39], and in our experiments, the percentage of B cells expressing IgD in the cell membrane was much lower than that of cells expressing IgM, suggesting that most of the liver IgM^+^ cells show undetectable levels of IgD on the cell surface. Taking this into account and knowing that B2-like responses in fish seem to be slower and less efficient than mammalian B2 lymphocyte responses, it would be very interesting to deeply characterize the nature of the resident IgM^+^ B cell subpopulation in the trout liver and whether they resemble mammalian B1-like lymphocytes.

Human hepatic T lymphocytes consist of a heterogeneous population of cells with different functional characteristics. In fact, conventional single positive CD4^+^CD8^-^ and CD8^+^CD4^−^ αβ T cells represent less than 40% of the isolated CD3^+^ hepatic cells, while in peripheral blood these single positive populations accounts for 70% of the CD3^+^ lymphocytes [4], [40]. In addition, the liver contains significant numbers of double negative (CD4^−^CD8^−^) T cells, double positive cells, cells bearing CD8αα chain homodimers, with no β chain association, γδTCR cells and cells that co-express an invariant TCR and NK-receptors (cells known as NKT lymphocytes). In our studies, CD8α^+^ cells constitute around 4% of the liver leukocyte population, but we can't provide information on the other T cell subpopulations because there are no other T lymphocyte markers available for rainbow trout. The high transcription levels of both CD4 molecules observed in rainbow trout liver, however, seem to suggest that cell bearing CD4 on the cell surface are also present in this organ.

In our viral model, the initial response to infection up-regulated mRNAs that seemed related to a T-dependent response, including CD3, CD8αβ, and CD4 genes. Furthermore, resting liver sinusoidal endothelial cells (LSEC) in rainbow trout appear to meet an important requirement necessary for extra-lymphatic priming of T cells (such as antigen-presenting capacity) and consequently could be priming naïve T cells outside a lymphatic environment such as they do in mammals [41]. However, an important difference has been found in the mammalian extralymphatic priming process: in contrast with the differentiation of naïve CD4^+^ T cells primed by bone-marrow-derived antigen presenting cells, the naïve CD4^+^ T cells activated *in vitro* by LSEC expressed IL-4 and IL-10, generating a regulatory T cell phenotype that could drive to a down-regulation of immune responses and induction of tolerance. In rainbow trout, we also observed an up-regulation of IL-10 transcription after 5 days of infection suggesting a certain degree of regulation in trout liver responses.

In mammals, signals from the diverse PRRs converge on two signaling pathways (reviewed in [42]). Thus, all TLRs but TLR3 signal via the adaptor protein MyD88 (myeloid differentiation factor-88) leading to NF-κB (nuclear factor kappa-light-chain-enhancer of activated B cells) activation. TLR3, on the other hand, recruits the adaptor protein TRIF (TIR-domain containing adaptor recruiting interferon-β) that ultimately causes phosphorylation and nuclear localization of IRF-3 (IFN regulatory factor 3), the transcription factor that drives the synthesis of type I IFN. Similarly, RIG-I like receptors induce IRF-3 activation via mitochondrial IPS-1 (IFN-β promoter stimulator 1) induction. These pathways are optimized such that PRRs engaged by bacterial products generally promote NF-κB activation, whereas those pathways activated by viral infection strongly induce IFN-β. After VHSV infection, a typical viral PPR profile was induced in the liver, which included the up-regulation of TLR3, TLR22 (fish-specific), MDA-5 and LGP-2. Even though mammalian TLR7 (as TLR8) is activated by synthetic antiviral imidazoquinoline compounds and is implicated in the recognition of ssRNA, the specific ligands triggering TLR7 in fish are still unknown [43]. However, TLR7 was also significantly up-regulated in trout liver, and this seems to correlate with studies performed with vesicular stomatitis virus (VSV), a mammalian rhabdovirus, known to be specifically recognized by TLR7 [44].

An important number of chemokines were strongly up- or down-regulated as a consequence of the infection, including up-regulation of CK3, CK10, CK12 and CXCL11_L1, and down-regulation of CK9 and CXCL14. Among the chemokines up-regulated, CK10 and CK12 showed the strongest up-regulation from the second day of infection. Several rainbow trout chemokines, including CK9, CK10, CK11 and CK12 are strongly expressed in rainbow trout mucosal tissues, and transcriptionally regulated following viral infections or DNA vaccination in gills, skin and different gut segments [26], [45]. In addition, the pattern of chemokine gene regulation observed in the systemic organs (spleen and kidney) seems to be specific for different viral infections [46], with a VHSV-specific pattern that includes CK1, CK3, CK5B, CK6, CK12, CXCL11_L1 and CXCL-F1 up-regulation. Interestingly, many of these chemokines were also modulated by VHSV in the liver and it seems probable that at least some of them are involved in the recruitment of CD8α^+^ cells observed.

As part of the innate immune response, altered levels of several plasma proteins known as acute phase response (APR) contribute to fight against infections and to recover homeostasis after infection or tissue injury. The liver is the main organ for the production of these APR plasma proteins and teleost hepatic APR proteins have been previously characterized in several studies [47]–[49]. Even if regulation of complement components, including C3, was reported in trout liver after bacterial challenges [50], [51], the transcription of the complement component C3 was not affected after viral infection in our studies. On the other hand, SAA was strongly up-regulated in response to VHSV in accordance to the induction previously observed in teleost liver in response to parasites, bacteria or LPS stimulation [52]–[55]. The strong induction of SAA after VHSV infection correlated with the induction of the inflammatory cytokine IL-6, but not with IL-1β, suggesting a well-defined anti-viral response instead of a general inflammatory reaction. Additionally, we observed strong induction of hepcidin in liver samples and no induction of LEAP-2a genes after VHSV infection. LEAP -1 (also known as hepcidin) and LEAP-2, are blood-derived antimicrobial peptides expressed predominantly in the liver of mammals [56], birds [57] and bony fish [58]–[60]. It has been already shown that hepcidin gene is strongly and differentially expressed in the liver after bacterial challenge in fish [58], while LEAP-2 peptides are induced in intestine and skin following bacterial exposure [60]. The induction of hepcidin again suggest a role of the teleost liver in the regulation of the immune response because hepcidin functions as a systemic iron-regulatory hormone that can be regulated in inflammatory processes, and can have a role also as an anti-inflammatory molecule [61], [62].

In summary, we can infer that a local, dynamic population of hepatic lymphocytes resides in the rainbow trout liver. Our results demonstrate that T lymphocytes play a key role in the initial response to VHSV in the liver, with an up-regulation of CD3, CD8, CD4, perforin, Mx and interferon (IFN) genes. Consequently, we observed a recruitment of CD8α^+^ cells in the liver in response to viral infection, whereas no differences were found in the percentages of IgM^+^ and IgD^+^ populations in response to the virus. In addition, the viral infection provoked the activation of several PRRs including TLR3, TLR7, TLR22, MDA5, and LGP2a and b. Regulation of chemokine transcription was also appreciated in the liver from day 2 post-infection, with an increase of CK10, CK12 and CXCL11-L1, and a decrease of CK9 and CXCL14. Our results point to the liver as an important site for immune regulation during the course of viral infections.

## Supporting Information

Figure S1 **Oligonucleotides used for real time PCR in this study.**
(DOCX)

Figure S2 **Specificity of the IgM and IgT Mabs used in immunohistochemistry.** One-dimensional sodium dodecyl sulfate-polyacrylamide gel electrophoresis (SDS-PAGE) and Western blotting were performed by standard protocols to validate the monoclonal mouse antibodies raised against trout IgM and IgT used in immunohistochemistry. Briefly described: rainbow trout spleen lysate was loaded in lanes 1, 4 and 6 on the SDS-PAGE gel. The recombinant protein with which the IgT antibodies were raised against (for a description of this see Olsen et al. 2011) were loaded in lanes 2 and 3. Rainbow trout plasma was loaded in lane 5. Lanes 1–5 were run under reduced conditions and lane 6 was run under non-reduced conditions (150 V, 1.5 h). After transfer of the proteins from the gel to a PVDF membrane lane 1, 2 and 6 were incubated overnight at 4°C in 1∶10 dilution of the mouse anti-trout IgT antibodies. Lanes 3–5 were incubated overnight at 4°C with a 1∶10 dilution of the mouse anti-trout IgM antibody (see Jørgensen *et al*. 2011 for a description of Mab production). After 3×10 min washes in standard washing buffer the membranes were incubated for 1 h at room temperature in 1∶1000 p260 (DAKO) secondary anti-mouse antibodies conjugated to HRP (Horse Radish Peroxidase). Subsequently, the blots were washed as previously described and then developed with 3,3′-diaminobenzidine (DAB). Results: lane 1 shows one band of approximately 66 kDa, which corresponds to the heavy chain of IgT (Zhang *et al*. 2010). Lane 2 shows a prominent band of around 42 kDa, which corresponds to the recombinant protein. The recombinant protein polymerizes into di- and trimers, which can be seen on the blot as weaker bands of approximately 84 kDa and 126 kDa. Lane 3 shows that IgM does not bind to the recombinant IgT peptide. Lane 4 and 5 shows the heavy chain of IgM from spleen and plasma respectively. Lane 6 shows IgT in the spleen under non-reduced conditions (Zhang *et al*. 2010).
(TIF)

Figure S3 **Constitutive transcription levels of immune genes in liver samples obtained from unstimulated perfused trout.** Data are shown as the mean gene expression relative to the expression of endogenous control EF-1α ± SD (N = 3 individual fish).
(TIF)

Figure S4 **Transcription of G VHSV gene in liver after intraperitoneal viral infection.** Rainbow trout were infected with VHSV as described in the legend of Figure 4 and the liver sampled to determine the levels of expression of the VHSV G gene by real-time PCR. Data are shown as the mean gene expression relative to the expression of endogenous control EF-1α ± SD (N = 6 individual fish).
(TIF)

Figure S5 **Imunohistochemical detection of different leukocyte populations in control and infected trout livers.** Photomicrographs of anti-IgM, anti-IgD, anti-IgT, anti-CD3, and anti-MHC-II positive staining in liver sections obtained from control and VHSV infected fish. Two representative photomicrographs are shown for each group. Counterstained with Mayer's haematoxylin. Scale bar represents 20 *µ*m.
(TIF)
