# Supplementary material for: Early Immune Responses in Rainbow Trout Liver upon Viral Hemorrhagic Septicemia Virus (VHSV) Infection
Source: PLoS One. 2014 Oct 22;9(10):e111084. doi: 10.1371/journal.pone.0111084 (PMC4206492; doi:10.1371/journal.pone.0111084)
Supplement: Figure S1 — Oligonucleotides used for real time PCR in this study. (DOCX) [file pone.0111084.s001.docx]

**Figure S1. Oligonucleotides used for real time PCR in this study.**

| **Gene** | **Primer** | **Sequence** |
| --- | --- | --- |
| EF1α | rtEF1α F | GATCCAGAAGGAGGTCACCA |
|  | rtEF1α R | TTACGTTCGACCTTCCATCC |
| CCR6 | CCR6 F | TGCAGAGGAAACAGTTAACAATTCA |
|  | CCR6 R | CCAGTAAACCCAGGATACAGATGAC |
| CCR7 | CCR7 4F | TTCACTGATTACCCCACAGACAATA |
|  | CCR7 4R | AAGCAGATGAGGGAGTAAAAGGTG |
| CCR9 | CCR9 F | TCAATCCCTTCCTGTATGTGTTTGT |
|  | CCR9 R | GTCCGTGTCTGACATAACTGAGGAG |
| CCR9B | CCR9B F | AATATTTCCAACGTCTGAAACAGGA |
|  | CCR9B R | CTCACCCAGGACTTATCACACATTC |
| CCR13 | CCR13 F | GTTCTGTACAACGTCTGGAAGGATT |
|  | CCR13 R | ATGGCCAAAGGAAGTAGAAAGAAGA |
| CXCR1 | CXCR1 F | CCTGATATCCAGAAGCTCTTTGTGT |
|  | CXCR1 R | TTGCATCCAGCTCTATGATAATGAA |
| CXCR3A | CXCR3A F | CAAGGCAACCACAAATTACTATATTTATGATG |
|  | CXCR3A R | CAGCACACACAGCACCAGGAT |
| CXCR3B | CXCR3B F | CACTGGAGCCATGTTTACAATCAACT |
|  | CXCR3B R | CCCTCACAGACTCCAGGAAGTG |
| CXCR4 | CXCR4 F | GTGCATGTGATCTACACCATC |
|  | CXCR4 R | GAGCTGTGGCAAACACTATGT |
| CK1 | CK1 F | GATGGCTGAAAGGCTACACC |
|  | CK1 R | TGGGATTTGTTCTCCTGACG |
| CK3 | CK3 F | AGATCACCGTTCCCATCATC |
|  | CK3 R | GTGACTTTCTGGCCATCTCC |
| CK5B | CK5B F | TTTGCTGATCGTCAGATACCC |
|  | CK5B R | GTGTCTGCTCCCCAGACTTC |
| CK6A | CK6A F | TGAAAGGCCTACGAATCTGC |
|  | CK6A R | GTTGTTGTTGGCTGGTTGTG |
| CK9 | CK9 F | AAGGCTCTTATGGGAACTGC |
|  | CK9 R | CCACTTCTGGCTGGGATTG |
| CK10 | CK10 F | ATTGCCAAGATCCTCTTCTGTGTTC |
|  | CK10 R | CCTGAGGCTGGTAACCTATGACAAC |
| CK11 | CK11 F | GAACATTCCTTTGAGCATACTAAT |
|  | CK11 R | TGCACAATACTTCCTCCCAT |
| CK12 | CK12 F | GACATCGATGCCACTGTGTT |
|  | CK12 R | GGAGATGGTTCGCTCCAGAC |
| CXCL8_L1 | CXCL8 F | ATTGAGACGGAAAGCAGACG |
|  | CXCL8 R | CTTGCTCAGAGTGGCAATGA |
| CXCL_F1 | CXCL F | GCTCACACTGCTCTAAGGAAGAA |
|  | CXCL R | GGAGAGAGTCTCAATGGAACGT |
| CXCL11_L1 | CXCL11 F | TGGACTGGTGAACCGTGTTA |
|  | CXCL11 R | TCTTGGCAAATGGAGCTTCT |
| CXCL12A | CXCL12A F | GACATACGTTCCTTTCGCACA |
|  | CXCL12A R | GCTTTCTTCATCTTGTTGAGGGCA |
| CXCL12B | CXCL12B F | CATGGATGTACCTCCCTCTACG |
|  | CXCL12B R | CTTCTTCACCTTGTTAATGGCA |
| CXCL14 | CXCL14 F | TCCCTACAAGCTGAAGCCTAC |
|  | CXCL14 R | TGCTTATCCTTCCAGATCCG |
| IgMH | rtIgMh F | TGCGTGTTTGAGAACAAAGC |
|  | rtIgMh R | GACGGCTCGATGATCGTAAT |
| secretedIgM | sIgM F | CCTTAACCAGCCGAAAGGG |
|  | sIgM R | TGAGGTTCTATCAATGGTTCTC |
| membraneIgD | mIgD F | CAGGAGGAAAGTTCGGCATCA |
|  | mIgD R | CCTCAAGGAGCTCTGGTTTGGA |
| secretedIgD | sIgD F | TGGCACGCCAGGATTTGAC |
|  | sIgD R | TCAGAATTGAGTGAACGGACAGACA |
| IgT | rtIgT F | AACATCACCTGGCACATCAA |
|  | rtIgT R | TTCAGGTTGCCCTTTGATTC |
| CD3 | CD3I F | CCTGATTGGAGTAGCTGTCTAC |
|  | CD3I R | GCTGTACTCAGATCTGTCCATGC |
| CD8α | rtCD8a F | ACGACTACACCAATGACCACAACC |
|  | rtCD8a R | CAGTGATGATGAGGAGGAGGAAGA |
| CD8β | rtCD8b F | GTTCAAGGCCAGTAAAAGGGACAT |
|  | rtCD8b R | GCCTCCACAACTCGTTCTCTTTCT |
| CD4-1 | rtCD4-1 F | GAGTACACCTGCGCTGTGGAAT |
|  | rtCD4-1 R | GGTTGACCTCCTGACCTACAAAGG |
| CD4-2a | rtCD4-2 F | GTGCTCATCGTGGATTATTTT |
|  | rtCD4-2 R | GGTGGACCTTGACTGTGACAAT |
| Blimp1 | Blimp1 F | AGCTGTCCAACCTCAAGGTCC |
|  | Blimp1 R | TTGCGGCACACCTGGGCATTC |
| Pax5 | Pax5 F | ACGGAGATCGGATGTTCCTCTG |
|  | Pax5 R | GATGCCGCGCTGTAGTAGTAC |
| Lamp3 | Lamp3 F | CATGAAAAGCTGTTCCCAACTGC |
|  | Lamp3 R | GCACTCAACCTCCTCTCCAAAC |
| TLR1 | TLR1 F | CAGACGCCCTGTTGATGTTC |
|  | TLR1 R | CCTTCACAAGTTCCACCACG |
| TLR2 | TLR2 F | GATCCAGAGCAACACTCTCAACAT |
|  | TLR2 R | CTCCAGACCATGAAGTTGACAAAC |
| TLR3 | TLR3 F | AGCCCTTTGCTGCCTTACAGAG |
|  | TLR3 R | GTCTTCAGGTCATTTTTGGACACG |
| TLR5 | TLR5 F | TTGACTTATCTTCCAACGGATTCA |
|  | TLR5 R | CTTTGAAATTGCTGAAACCAAATG |
| TLR7 | TLR7 F | TACAGCTTGGTAACATGACTCTCC |
|  | TLR7 R | CAACTCTCTGAGACTTGTCGGTAA |
| TLR8a2 | TLR8 F | CATCTATGTTCTCATCCAGCAACC |
|  | TLR8 R | GGTCCCCCTAATAGACAACCTCTT |
| TLR9 | TLR9 F | TCTTCATAGAGCTGAAGAGGCCTCA |
|  | TLR9 R | GTTCCCACTGAGGAGAAGTGTTTT |
| TLR22 | TLR22 F | AGTGGACAATGACGCTCTTTTAC |
|  | TLR22 R | GAGCTGATGGTTGCAATGAGG |
| MDA5 | MDA5 F | AGAGCCCGTCCAAAGTGAAGT |
|  | MDA5 R | GTTCAGCATAGTCAAAGGCAGGTA |
| LGP2a | LGP2a F | ACACCTGCTCTTTCCGTCAC |
|  | LGP2a R | GTTGGCTGGATGTCCTTTGG |
| LGP2b | LGP2b F | GTGGCAGGCAATGGGGAATG |
|  | LGP2b R | CCTCCAGTGTAATAGCGTATCAATCC |
| C3 | C3 F | GAGATGGCCTCCAAGAAGATAGAA |
|  | C3 R | ACCGCATGTACGCATCATCA |
| Serum amyloid A | SAA F | GGTGAAGCTGCTCAAGGTGCTAAAG |
|  | SAA R | GCCATTACTGATGACTGTTGCTGC |
| Hepcidin | Hepcidin F | GCTGTTCCTTTCTCCGAGGTGC |
|  | Hepcidin R | GTGACAGCAGTTGCAGCACCA |
| LEAP 2A | LEAP F | GGTTCCTGGTGTTTCTGGTGCT |
|  | LEAP R | AGTGGCCACCCCTGCAAAT |
| totalMx | tMx F | AGCTCAAACGCCTGATGAAG |
|  | tMx R | ACCCCACTGAAACACACCTG |
| IFNI | IFNI F | AAAACTGTTTGATGGGAATATGAAA |
|  | IFNI R | CGTTTCAGTCTCCTCTCAGGTT |
| IL1β | IL1β F | GACATGGTGCGTTTCCTTTT |
|  | IL1β R | ACCGGTTTGGTGTAGTCCTG |
| IL6 | IL6 F | CCTTGCGGAACCAACAGTTTG |
|  | IL6 R | CCTCAGCAACCTTCATCTGGTC |
| IL10 | IL10 F | CTGCTGGACGAAGGGATTCTAC |
|  | IL10 R | GGCCTTTATCCTGCATCTTCTC |
| Perforin 1 | Perforin F | GGAACGACGACCTGTTAGGA |
|  | Perforin R | TCATAGGGGAGGGCACATAG |
| VHSV-G | VHSVG F | AAGGATCACGAGTACCCGTTCTTC |
|  | VHSVG R | CCCAATAGACTCCCTGCCAATG |
