# Supplementary figures and images for: Early Immune Responses in Rainbow Trout Liver upon Viral Hemorrhagic Septicemia Virus (VHSV) Infection
Source: PLoS One. 2014 Oct 22;9(10):e111084. doi: 10.1371/journal.pone.0111084 (PMC4206492; doi:10.1371/journal.pone.0111084)

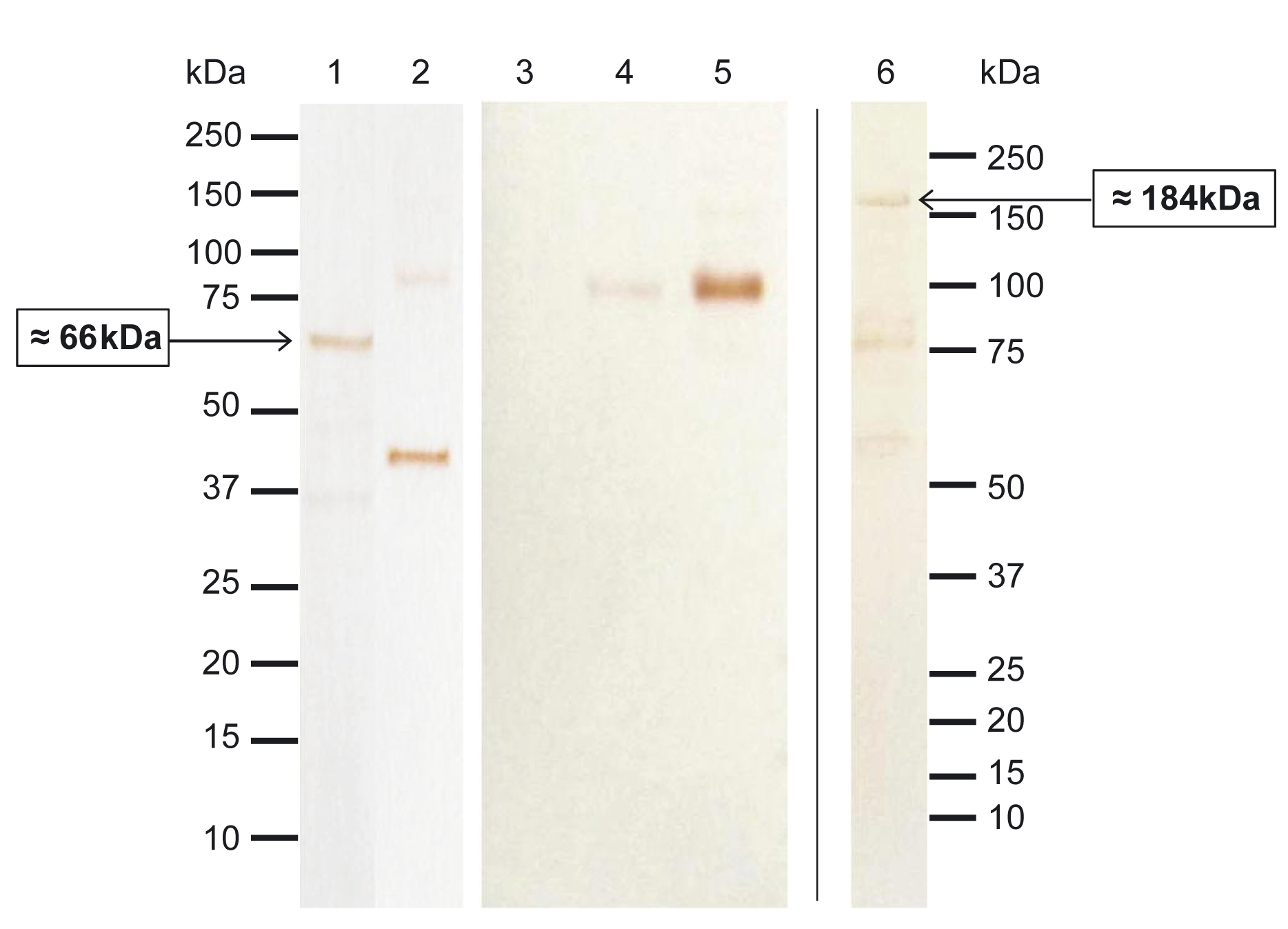

Supplement: Figure S2 — Specificity of the IgM and IgT Mabs used in immunohistochemistry. One-dimensional sodium dodecyl sulfate-polyacrylamide gel electrophoresis (SDS-PAGE) and Western blotting were performed by standard protocols to validate the monoclonal mouse antibodies raised against trout IgM and IgT used in immunohistochemistry. Briefly described: rainbow trout spleen lysate was loaded in lanes 1, 4 and 6 on the SDS-PAGE gel. The recombinant protein with which the IgT antibodies were raised against (for a description of this see Olsen et al. 2011) were loaded in lanes 2 and 3. Rainbow trout plasma was loaded in lane 5. Lanes 1–5 were run under reduced conditions and lane 6 was run under non-reduced conditions (150 V, 1.5 h). After transfer of the proteins from the gel to a PVDF membrane lane 1, 2 and 6 were incubated overnight at 4°C in 1∶10 dilution of the mouse anti-trout IgT antibodies. Lanes 3–5 were incubated overnight at 4°C with a 1∶10 dilution of the mouse anti-trout IgM antibody (see Jørgensen et al. 2011 for a description of Mab production). After 3×10 min washes in standard washing buffer the membranes were incubated for 1 h at room temperature in 1∶1000 p260 (DAKO) secondary anti-mouse antibodies conjugated to HRP (Horse Radish Peroxidase). Subsequently, the blots were washed as previously described and then developed with 3,3′-diaminobenzidine (DAB). Results: lane 1 shows one band of approximately 66 kDa, which corresponds to the heavy chain of IgT (Zhang et al. 2010). Lane 2 shows a prominent band of around 42 kDa, which corresponds to the recombinant protein. The recombinant protein polymerizes into di- and trimers, which can be seen on the blot as weaker bands of approximately 84 kDa and 126 kDa. Lane 3 shows that IgM does not bind to the recombinant IgT peptide. Lane 4 and 5 shows the heavy chain of IgM from spleen and plasma respectively. Lane 6 shows IgT in the spleen under non-reduced conditions (Zhang et al. 2010). (TIF) [file pone.0111084.s002.tif]

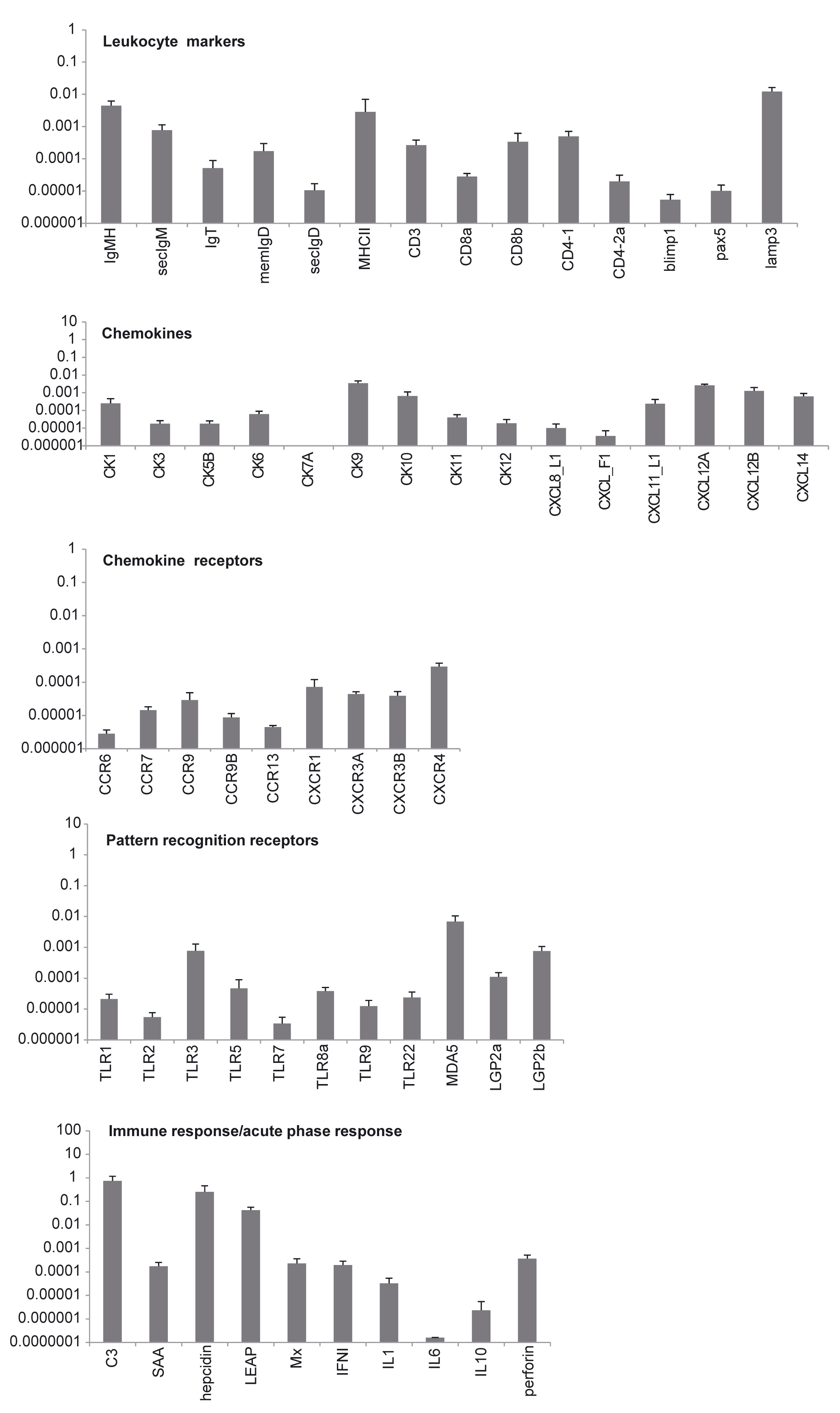

Supplement: Figure S3 — Constitutive transcription levels of immune genes in liver samples obtained from unstimulated perfused trout. Data are shown as the mean gene expression relative to the expression of endogenous control EF-1α ± SD (N = 3 individual fish). (TIF) [file pone.0111084.s003.tif]

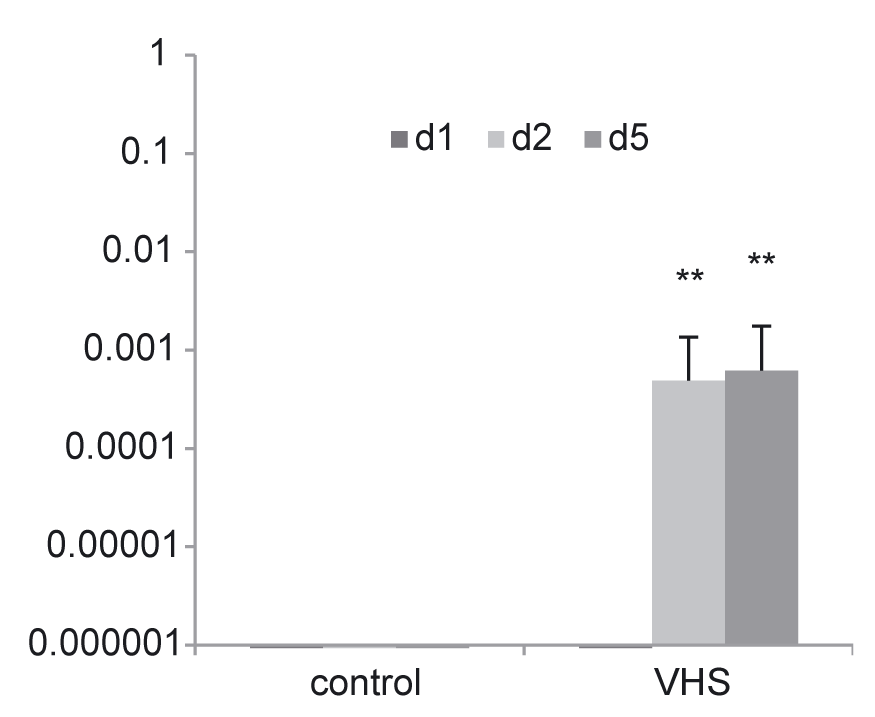

Supplement: Figure S4 — Transcription of G VHSV gene in liver after intraperitoneal viral infection. Rainbow trout were infected with VHSV as described in the legend of Figure 4 and the liver sampled to determine the levels of expression of the VHSV G gene by real-time PCR. Data are shown as the mean gene expression relative to the expression of endogenous control EF-1α ± SD (N = 6 individual fish). (TIF) [file pone.0111084.s004.tif]

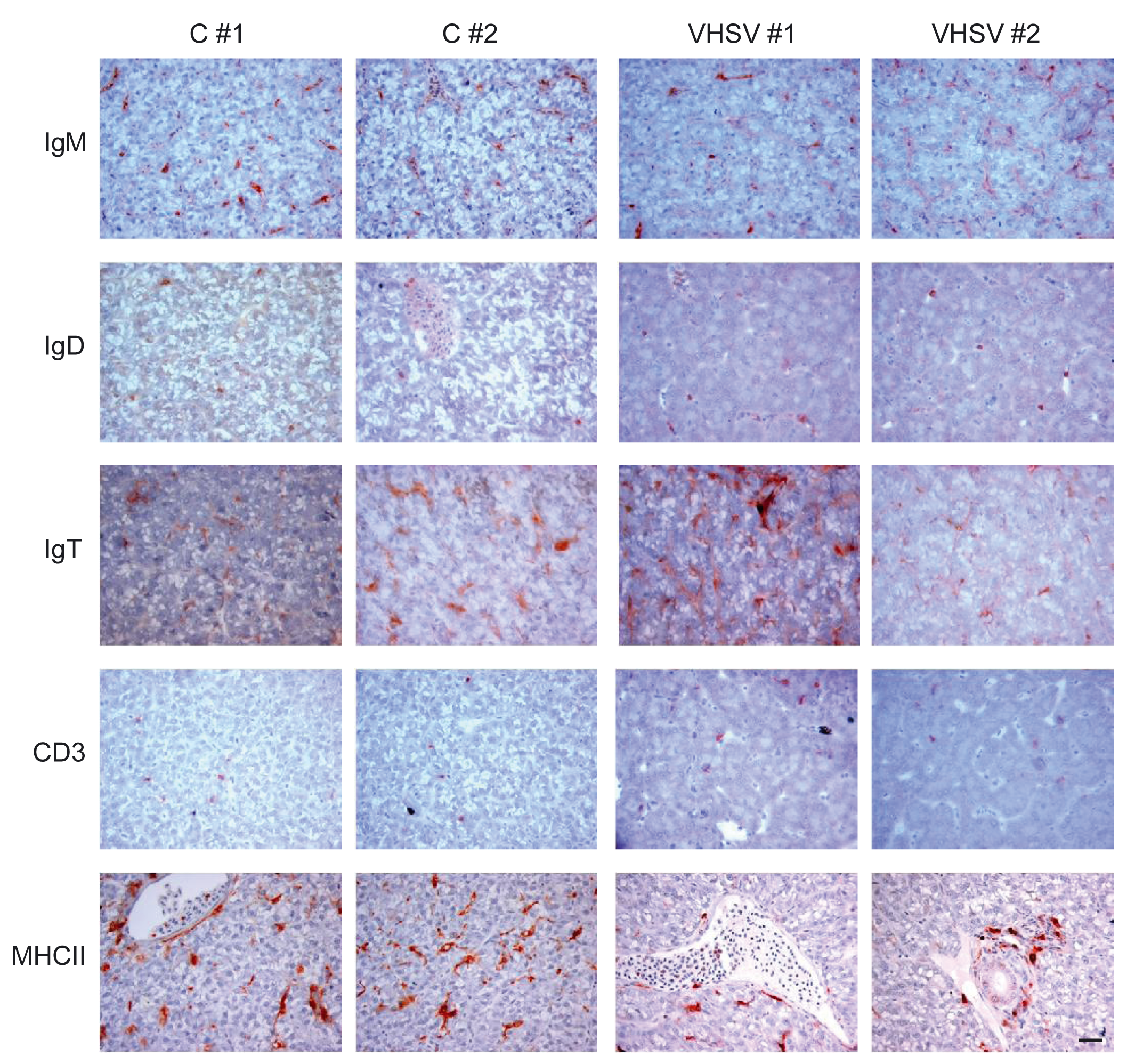

Supplement: Figure S5 — Imunohistochemical detection of different leukocyte populations in control and infected trout livers. Photomicrographs of anti-IgM, anti-IgD, anti-IgT, anti-CD3, and anti-MHC-II positive staining in liver sections obtained from control and VHSV infected fish. Two representative photomicrographs are shown for each group. Counterstained with Mayer's haematoxylin. Scale bar represents 20 µm. (TIF) [file pone.0111084.s005.tif]
